# Anti-leukemia effects of the novel synthetic 1-benzylindole derivative 21-900 *in vitro* and *in vivo*

**DOI:** 10.1038/srep42291

**Published:** 2017-02-09

**Authors:** Wei-Chun HuangFu, Min-Wu Chao, Chun-Chun Cheng, Yu-Chieh Wei, Yi-Wen Wu, Jing-Ping Liou, George Hsiao, Yu-Ching Lee, Chia-Ron Yang

**Affiliations:** 1 The Ph.D. Program for Cancer Biology and Drug Discovery, College of Medical Science and Technology, Taipei Medical University, Taipei, Taiwan; 2 Graduate Institute of Medical Sciences, School of Medicine, Taipei Medical University, Taipei, Taiwan; 3 School of Pharmacy, College of Medicine, National Taiwan University, Taipei, Taiwan; 4 School of Pharmacy, College of Pharmacy, Taipei Medical University, Taipei, Taiwan; 5 Department of Pharmacology, School of Medicine, Taipei Medical University, Taipei, Taiwan; 6 The Center of Translational Medicine, Taipei Medical University, Taipei, Taiwan; 7 The Ph.D. Program for Medical Biotechnology, College of Medical Science and Technology, Taipei Medical University, Taipei, Taiwan

## Abstract

Cancers are the major cause of death worldwide. Chemotherapy using cytotoxic drugs and targeted therapy is required when surgery is difficult, ineffective, or impossible. We previously synthesized the novel synthetic 1-benzylindole derivative 21-900 and found that it inhibits histone deacetylase (HDAC) activities and tubulin assembly. Here we tested its effects on the human leukaemia cell lines HL-60 and MOLT-4 *in vitro* and *in vivo*. We found that its potent cytotoxic effects were mediated through cell cycle arrest at the G2/M phase, which increased the population of sub-G1 cells, leading to apoptosis. Further, tubulin was depolymerized by 21-900 in a manner similar to that of vincristine, leading to disruption of microtubule dynamics and increased levels of the mitotic marker MPM-2. Further, 21-900 increased the expression of cleavage form of poly (ADP-ribose) polymerase (PARP), caspase 3, 7 (cleavage form), and pro-apoptotic protein BAK and decreased the expression of pro-survival BCL-2-family proteins BCL-2, MCL-1, and BID pro-form, leading to the induction of apoptosis. The growth of tumours in nude mice formed by xenografts of HL-60 and MOLT-4 cells was significantly inhibited by 21-900 without causing the mice to lose body weight. These findings indicate that 21-900 may serve as a potent anti-leukaemia drug.

Microtubules form heterodimers comprising α- and β-tubulins, which are dynamic structures that play an important role in cellular processes such as cell division^1^. During the cell cycle, microtubules form an intracellular lattice-like structure, and when cells enter mitosis, the original reticular microtubules are remodelled and assembled into mitotic spindles that attach to and separate chromosomes^2^. Microtubule-targeting agents (MTAs) that interfere with microtubule dynamics inhibit the ability of cells to complete mitosis, which limits cell proliferation^3^. Therefore, MTAs have been used as anti-cancer drugs for the past two decades^4^. MTAs are classified as agents that destabilise (e.g. vincristine) or stabilise (e.g. paclitaxel) microtubules^4^. At lower concentrations, both types of MTAs affect only microtubule dynamics, which causes abnormal mitotic spindle formation, cell-cycle arrest in M phase, and subsequent apoptosis^5^. Although these agents have achieved notable clinical success due to arrest of cell division in metaphase, adverse effects associated with drug resistance and serious toxicities, such as myelosuppression, peripheral sensory neuropathy, as well as the drugs’ poor solubility, remain to be addressed^6^. Therefore, continued efforts are required to develop MTAs that act with greater efficacy and reduced adverse effects.

Histone deacetylases (HDACs) deacetylate the lysine residues of histones or non-histone proteins and mediate epigenetic regulation through post-translational modifications of their substrates^7^. The biological activities of different HDACs differ because of the distribution of their diverse substrates. For example, HDAC6 predominately resides in the cytoplasm, where it deacetylates tubulins, and the close linkage of acetylation with microtubule stability^8^,^9^ suggests that it plays pivotal roles in microtubule dynamics^10^,^11^.

HDAC overexpression has been observed in many cancers and is, therefore, considered new target of cancer therapy; indeed, four HDAC inhibitors are approved for clinical use in T-cell lymphoma and multiple myeloma^12^. We synthesised a series of 1-benzylindoles derivatives based on a combination of the structures of tubulins and HDAC inhibitors. 21-900 served as a potent anticancer agent with dual tubulin and HDAC inhibitory activity, thereby exhibiting the most potent anti-proliferation and tubulin depolymerisation effects among the 1-benzylindoles derivatives tested^13^. However, we did not determine the mechanism by which 21-900 inhibits cancer growth. The purpose of the present study was to evaluate potential anti-cancer mechanisms and effects of 21-900 in *in vitro* and *in vivo* models of human leukaemia.

## Results

### HDACs activity and the growth of human leukemia cell lines are inhibited by 21-900

**Figure 1:**
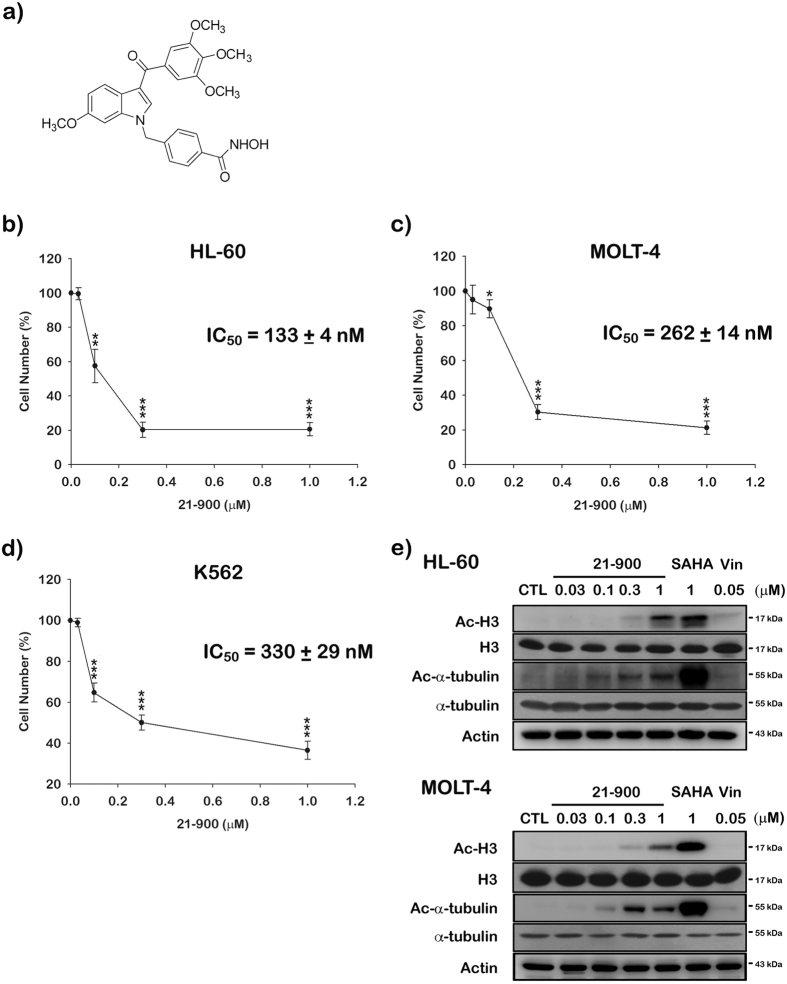
21-900 is cytotoxic to human leukaemia cell lines and potently inhibits HDACs. (**a**) Structure of 21-900. (**b**–**d**) The human leukaemia cell lines HL-60 (**b**), MOLT-4 (**c**), and K562 (**d**) (1 × 10^4^) were incubated with or without the indicated concentrations of 21-900 for 48 h, and cell viability was measured using the MTT assay. The results represent the mean ± SEM of three independent experiments at *p < 0.05, **p < 0.01, and ***p < 0.001 compared with untreated cells. (**e**) HL-60 and MOLT-4 cells (1 × 10^6^) were treated with the indicated concentration of 21-900, SAHA (1 μM) and vincristine (Vin) (0.05 μM) for 24 h. Whole-cell extracts were subjected to western blotting.

The structure of 21-900 is shown in Fig. 1a. We previously demonstrated that compared with other derivatives, 21-900 more effectively inhibits HDACs activity and the growth in A549, PC3 and HCT116 three different cancers cell lines^13^. Further, 21-900 was a more potent inhibitor of the growth of three types of cancer cell lines compared with the pan-HDAC inhibitor suberoylanilide hydroxamic acid (SAHA, Vorinostat)^13^. In particular, 21-900 most potently inhibited the proliferation of HL-60 cells (13, Fig. 1b–d). Therefore, we used human leukemia cell lines to assess potential anti-cancer effects of 21-900.

We evaluated the cytotoxic effects of 21-900 using the following leukaemia cell lines: human acute myeloid leukaemia (AML) HL-60, human acute lymphoblastic leukaemia (ALL) MOLT-4, and human chronic myeloid leukaemia (CML) K562 cells. Treating these cell lines with 21-900 reduced their viability (determined by MTT assay), indicated by the following IC_50_ values: 133 ± 4 nM, 262 ± 14 nM, and 330 ± 29 nM, respectively (Fig. 1b–d). Further, we found previously that 21-900 inhibits class I (HDAC1, 2) and class II (HDAC6) HDAC isoforms; however, 21-900 most effectively inhibits the activity of HDAC6 (class IIb) (IC_50_ = 64.5 ± 2.44 nM), which is similar to the inhibitory effect of SAHA (72.34 ± 1.89 nM)^13^. Biomarkers of HDAC inhibition, such as hyperacetylated histone 3 and α-tubulin, were detected in HL-60 and MOLT-4 cells (Fig. 1e) treated with 21-900, indicating that 21-900 is an HDAC inhibitor.

### The growth of human leukemia cell lines cultured in the presence of 21-900 arrests at the G2/M phase of the cell cycle followed by apoptosis

**Figure 2:**
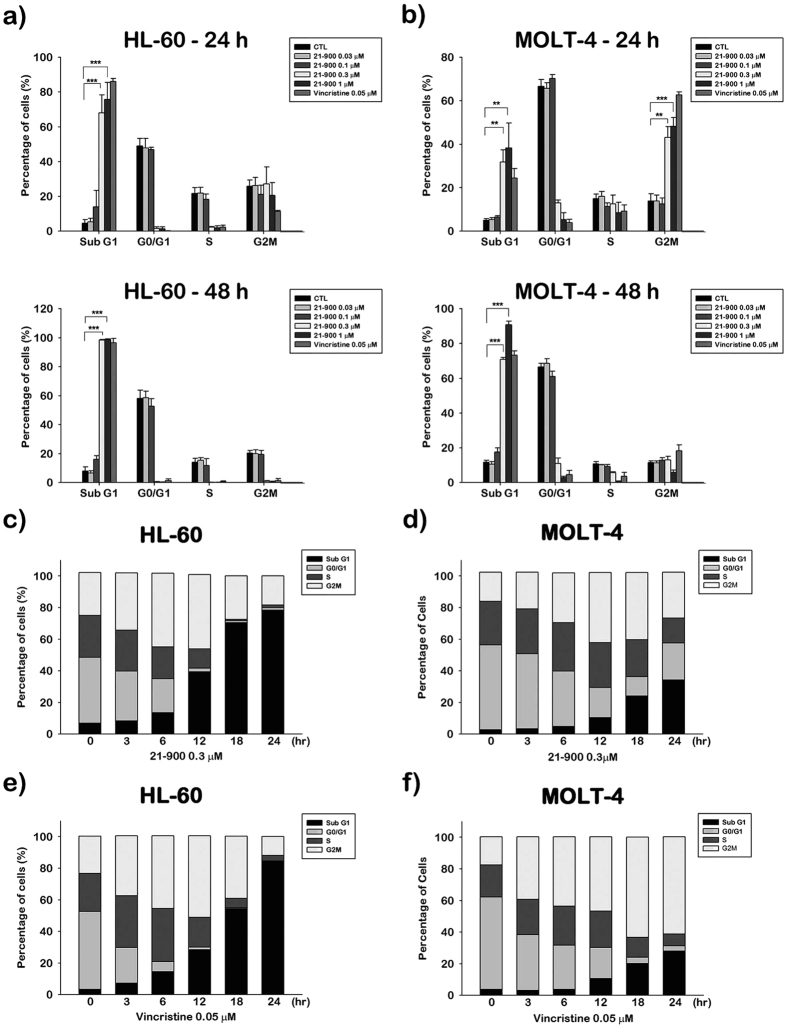
21-900 induces G2/M arrest and apoptosis of human leukaemia cell lines. (**a**) HL-60 or (**b**) MOLT-4 cells were treated with the indicated concentrations of 21-900 for 24 or 48 h, the cells were treated with propidium iodide, and the cell cycle was analysed using flow cytometry. The results represent the mean ± SEM of three independent experiments at *p < 0.05, **p < 0.01, and ***p < 0.001 compared with untreated cells. (**c** and **e**) HL-60 or (**d** and **f**) MOLT-4 cells were treated with 21-900 (3 μM) or vincristine (0.05 μM) for the indicated times, the cells were treated with propidium iodide, and the cell cycle was analysed using flow cytometry. The results represent three independent experiments.

We next investigated the effect of 21-900 on the cell cycle. In the presence of 21-900 for 24 h, HL-60 cultures accumulated cells with a sub-G1 DNA content, which indicates the induction of apoptosis (Fig. 2a). There was a further increase in the sub-G1 population after 48 h of treatment (Fig. 2a). By contrast, treatment of MOLT-4 cells with 21-900 for 24 h increased the G2/M population and the sub-G1 DNA content, and the sub-G1 population increased further after 48 h of treatment (Fig. 2b). The representative histograms of cell cycle distribution were shown in Supplemental Fig. 1. Further, treatment of HL-60 (Fig. 2c) and MOLT-4 cells (Fig. 2d) with 21-900 for 12 h induced cell cycle arrest in the G2/M phase, and the sub-G1 population subsequently increased. Vincristine treatment of HL-60 cells for 12 h induced significant cell cycle arrest in the G2/M phase (Fig. 2e) and that of MOLT-4 cells after 18–24 h (Fig. 2f), which was followed by an increase in the population of sub-G1 cells.

### Effect of 21-900 on mitotic arrest in human leukemia cells

**Figure 3:**
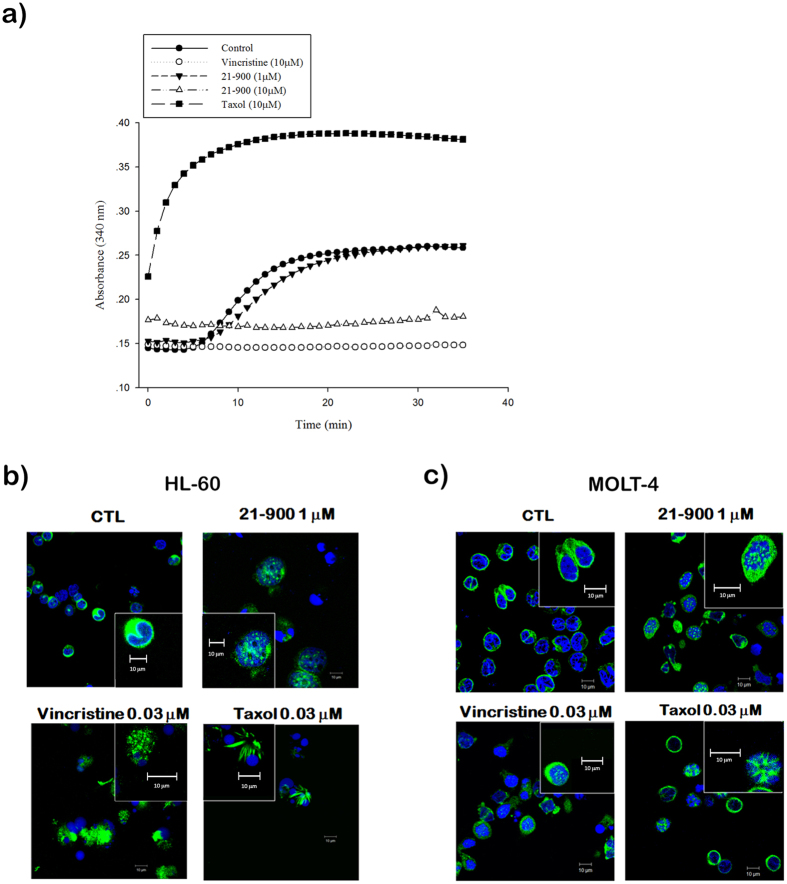
21-900 inhibits tubulin polymerization. (**a**) Tubulin assembly was determined using an *in vitro* tubulin polymerization kit. Cells were treated with vehicle (DMSO), 21-900 (1, 10 μM), vincristine (10 μM), or paclitaxel (10 μM) and analysed using spectrophotometry. (**b**) HL-60 and MOLT-4 cells were incubated with DMSO, 21-900 (1 μM), vincristine (0.03 μM), or paclitaxel (0.03 μM) for 24 h and incubated with an anti-β-tubulin antibody and DAPI. The microtubule network was analysed using a ZEISS LS 510 META confocal microscopy. Magnification: 600×. Inserts are enlarged 4×.

**Figure 4:**
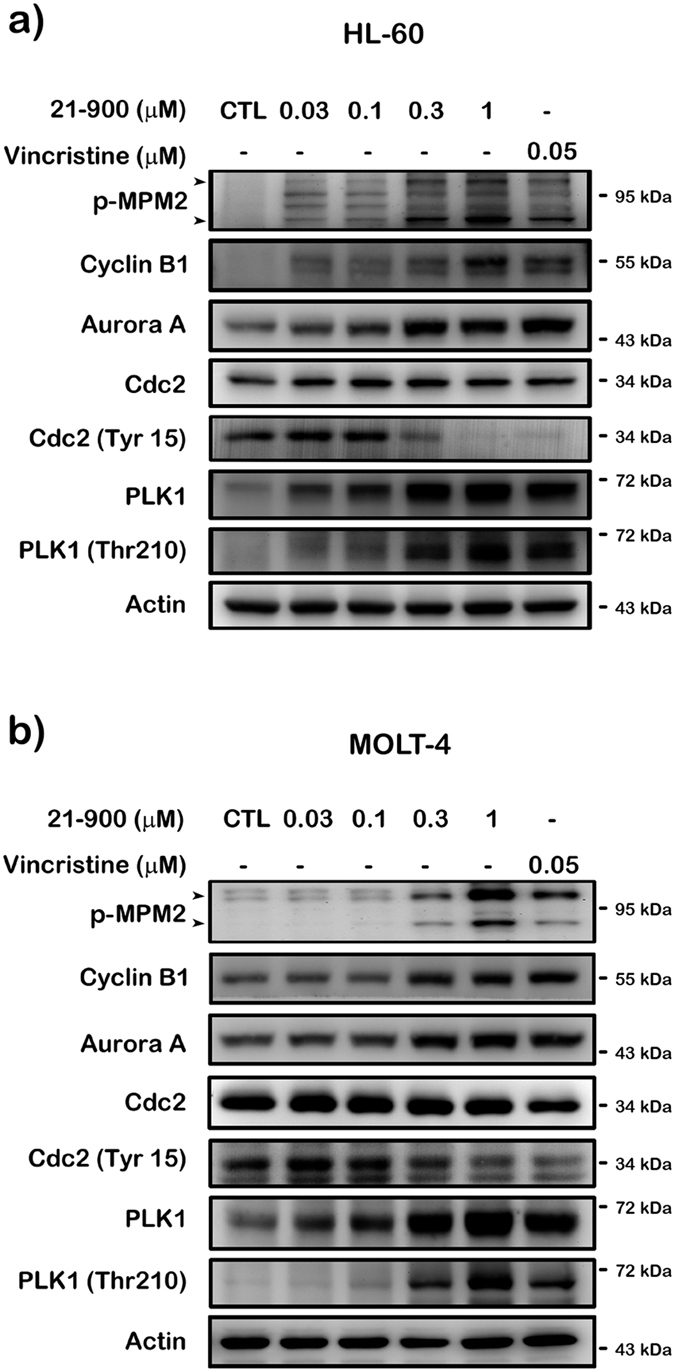
21-900 alters the levels of proteins that regulate the G2/M transition. (**a**) HL-60 or (**b**) MOLT-4 cells (1 × 10^6^) were incubated for 12 h with or without the indicated concentration of 21-900 or vincristine, and whole-cell lysates were subjected to western blotting with the indicated antibodies. Results represent three independent experiments.

Alteration of microtubule dynamics is a major mechanism that induces mitotic arrest. Therefore, we investigated whether 21-900 influenced microtubule dynamics. As shown in Fig. 3a, tubulin polymerises in a time-dependent manner in the presence of GTP at 37 °C (control group); 21-900 (10 μM) induced significant microtubule depolymerisation, similar to the effect of vincristine (10 μM). In contrast, the microtubule-stabilising agent paclitaxel (10 μM) induced a marked increase in microtubule polymerisation (Fig. 3a). Immunofluorescence analysis revealed the distribution of microtubules and the morphologies of HL-60 and MOLT-4 cells treated with 21-900, vincristine, or Taxol. As shown, microtubule depolymerisation in cells treated with 21-900 was similar to that induced by vincristine, indicating a diffusion of tubulin-specific fluorescence throughout the cytoplasm (Fig. 3b,c and Supplemental Fig. 2). We next determined the levels of G2/M phase regulatory proteins in cells treated with 21-900. We detected increased levels of mitosis-specific phosphorylated MPM2, cyclin B1, serine/threonine kinases polo-like kinase 1 (PLK1), p-Thr210 PLK1, and aurora kinase A and decreased levels of p-Tyr15 Cdc2 in both HL-60 (Fig. 4a) and MOLT-4 cells (Fig. 4b). These changes were similar to those induced by vincristine (Fig. 4a and b). These findings demonstrate that 21-900 induced G2/M phase arrest as well as microtubule dynamics instability.

### Effects of 21-900 on the apoptotic pathway in human leukemia cells

**Figure 5:**
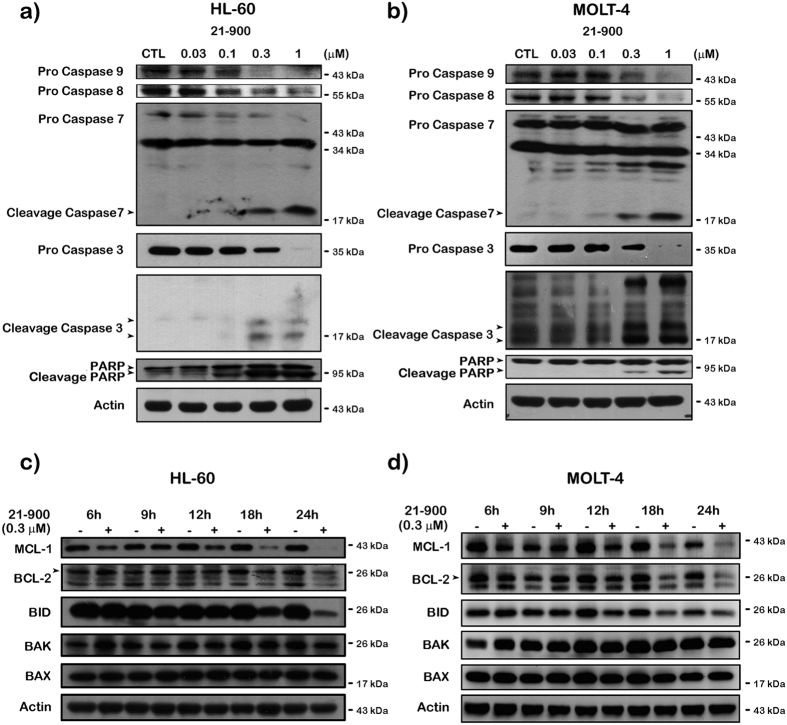
21-900 treatment induces apoptosis of HL-60 and MOLT-4 cells. (**a** and **c**) HL-60 or (**b** and **d**) MOLT-4 cells (1 × 10^6^) were incubated for 24 h or the indicated times with or without 21-900 (0.03–1 μM), and total cell lysates were subjected to western blotting. The results represent three independent experiments.

Our findings that the sub-G1 DNA content of human leukaemia cell lines significantly increased in cells treated with 21-900 led us to investigate the underlying mechanism. We found that in human leukaemia cell lines, expression of pro-caspases 3, 8, and 9 decreased and that of the cleaved forms of caspases 3, 7, and poly (ADP-ribose) polymerase (PARP) increased (Fig. 5a and b). The expression of the pro-survival members of the BCL-2 family BCL-2, MCL-1, and BID pro-form decreased in HL-60 (Fig. 5c) and MOLT-4 cells (Fig. 5d). However, the levels of pro-apoptotic proteins such as BAX and BAK were no responses to 21-900 treatment in both cells.

**Figure 6:**
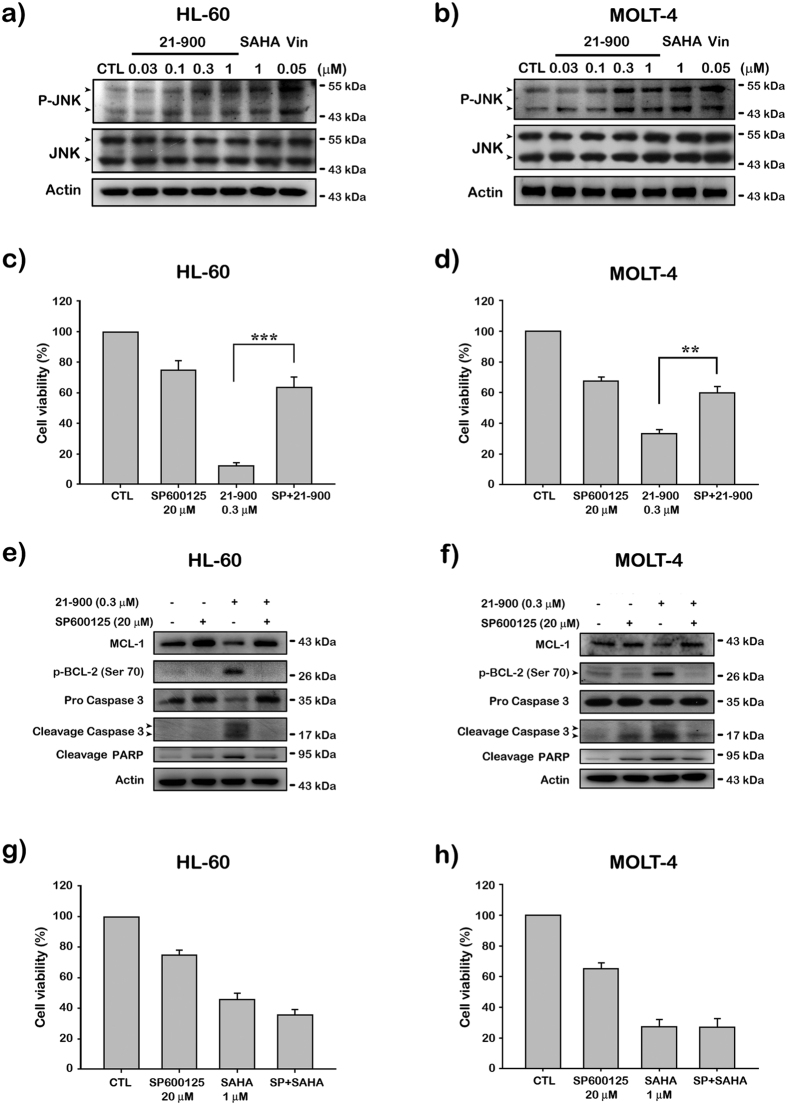
JNK activation mediates apoptosis induced by 21-900. (**a** and **e**) HL-60 or (b and f) MOLT-4 cells (1 × 10^6^) were treated with the indicated concentrations of 21-900, SAHA, vincristine, and SP600125 for 24 h. Whole-cell lysates were subjected to western blotting. (**c** and **g**) HL-60 or (**d** and **h**) MOLT-4 cells (3 × 10^5^) were incubated with 21-900 (0.3 μM) or SAHA (1 μM) with or without SP600125 (20 μM) for 48 h. Cell viability was measured using the MTT assay. The results represent the mean ± SEM of three independent experiments at *p < 0.05, **p < 0.01, and ***p < 0.001 compared with indicated groups.

JNK phosphorylation regulates cell apoptosis^14^. Therefore, we analysed the activation of JNK in cells treated with 21-900. JNK was activated in HL-60 (Fig. 6a) and MOLT-4 (Fig. 6b) cells treated with 21-900 in a dose-dependent manner, and 0.3 μM 21-900 significantly reduced the viability of both cell lines (Fig. 6c and d). By contrast, the JNK inhibitor SP600125 markedly reversed 21-900-induced apoptosis in both cells; similar result was also observed in the other specific JNK inhibitor JNK-IN-8 (Supplemental Fig. 3b), suggesting that JNK plays an important role in 21-900-induced apoptosis. Moreover, SP600125 nullified the effects of 21-900 on the levels of the cleaved forms of caspase 3, the decrease of MCL-1 levels, and the effects on BCL-2 phosphorylation (Fig. 6e and f). By contrast, SP600125 did not reverse the reduction in cell viability induced by SAHA (Fig. 6g and h).

### The survival of mice engrafted with human leukemia cells is prolonged by 21-900 through the inhibition of tumor growth

**Figure 7:**
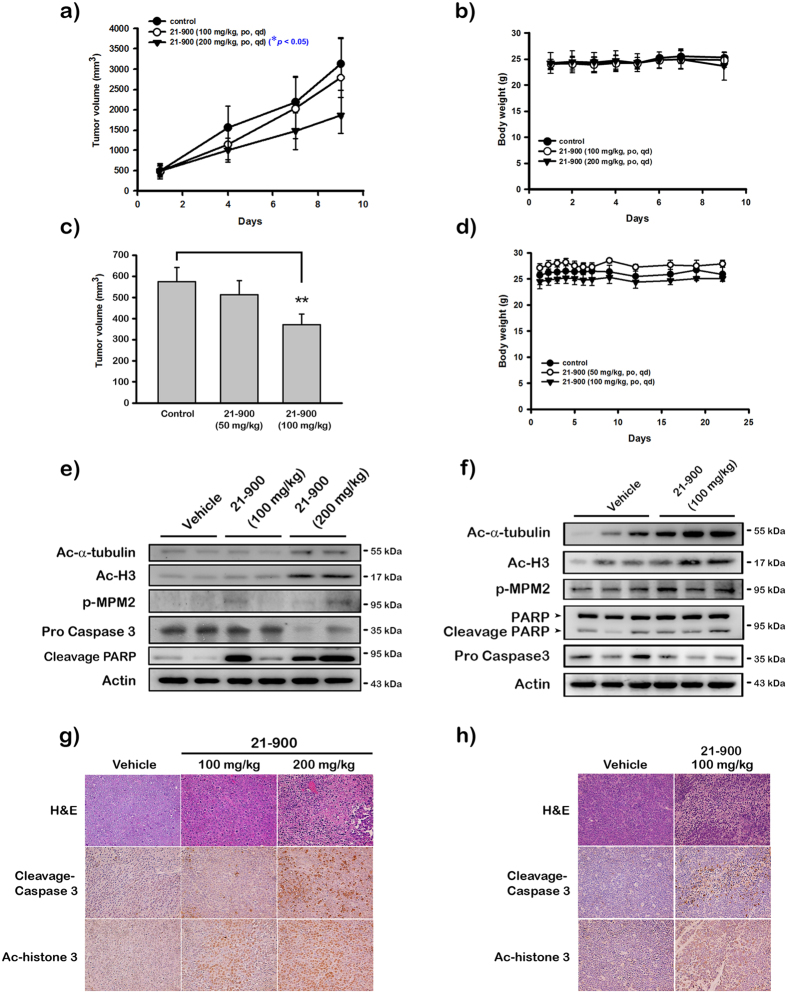
21-900 inhibits the growth of tumours in mice induced by engrafted human leukaemia cell lines and induces caspase 3 and PARP activation. Female SCID mice bearing tumours induced by HL-60 (**a** and **b**) or MOLT-4 (**c** and **d**) (~500 mm^3^) were divided into three groups (5 mice for each group) and treated 21-900 by orally, qd. The tumour volumes (**a** and **c**) and body weights (**b** and **d**) of mice were measured. Results represent the mean ± SEM at *p < 0.05, **p < 0.01 compared with controls (*n* = 5). (**e** and **f**) Once average size of the tumor volume was greater than 2,500 mm^3^, mice were sacrificed and tumors were resected. HL-60 (**e**) or MOLT-4 (**f**) xenograft tumour homogenates were analysed using western blotting. (**g** and **h**) Paraffin sections of HL-60 (**g**) or MOLT-4 (**h**) xenografts were stained with hematoxylin and eosin, an antibody against the cleaved form of caspase 3, or acetyl-histone H3. Sections were examined using light microscopy (400 x magnification).

The induction of apoptosis by 21-900 *in vitro* led us to determine its effects on tumours induced by human leukaemia cell lines in a mouse xenograft model. We found that administration of 21-900 (200 mg/kg) significantly reduced the volumes of tumours formed by HL-60 and MOLT-4 cells compared with controls (Fig. 7a and c). Further, the mice did not experience a significant loss of body weight (Fig. 7b and d). Moreover, western blotting and immunohistochemical analyses revealed that the expression of pro-caspases 3 decreased, cleaved forms of caspase 3 and PARP increased as well as the levels of MPM2, Ac-α-tubulin, and Ac-Histone 3 (Fig. 7e–7h). These results show that 21-900 treatment significantly inhibited tumour growth *in vivo*.

**Figure 8:**
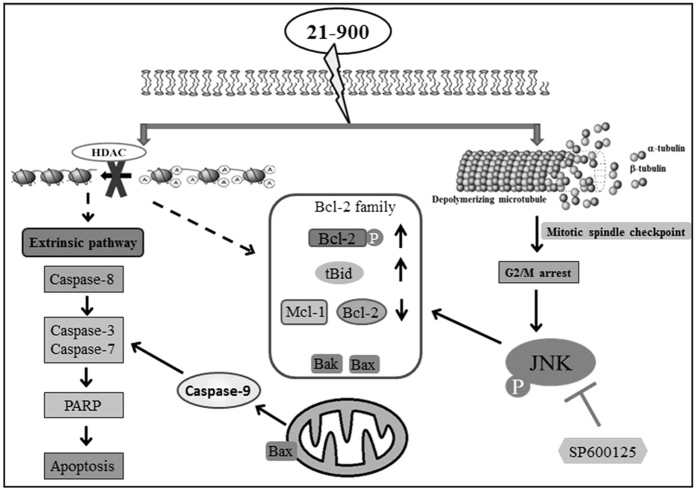
Summary of the mechanism of apoptosis induced by 21-900.

Together, our results show that 21-900 treatment significantly inhibited the growth of tumours formed by HL-60 and MOLT-4 cells and induced apoptosis *in vitro* and *in vivo*. Summary of apoptotic mechanism induced by 21-900 was illustrated in Fig. 8.

## Discussion

Current therapy for most types of acute leukaemia employs cytotoxic drugs and MTAs with impressive clinical outcomes^4^. However, drug resistance and neuronal toxicity are serious clinical problems^6^. To increase the value of chemotherapeutic agents, targeting a combination of specific motifs exhibited by different classes of molecules is a practical strategy employed in drug development^15^. Recent studies revealed a single treatment with HDAC inhibitors used in combination with conventional chemotherapeutic agents exhibits significant anti-cancer effects^16^,^17^, and increasing evidence suggests that HDAC inhibitors combined with MTAs synergise to inhibit cell proliferation and induce apoptosis^17^. Thus, a combined treatment that includes MTAs to target distinct molecules such as HDACs may show potential as a strategy for treating cancers. For example, HDACs are a common target used to validate this strategy^15^. Accordingly, the novel 1-benzylindole derivative 21-900 was synthesised by incorporating hydroxamic acid-containing moieties that inhibit HDAC activity in combination with the tubulin assembly inhibitor BPR0L075 in a phase II trial^18^. Here we show that 21-900 induced cell-cycle arrest at G2/M, depolymerisation of microtubules, and, subsequently, apoptosis of the human leukaemia cell lines HL-60 and MOLT-4. Further, we employed a xenograft model to show that 21-900 significantly inhibited the growth of tumours induced by engrafted HL-60 and MOLT-4 cells. The mice did not lose body weight, suggesting that 21-900 may serve as an anti-cancer agent.

To identify the mechanism of action of 21-900, we treated HL-60 and MOLT-4 cells with 21-900. These experiments revealed that treatment with 21-900 significantly increased the acetylation of histone H3 and α-tubulin (Fig. 1e). HDAC inhibitors induce apoptosis through a caspase-dependent pathway^19^,^20^. We show here that 21-900 increased in the population of cells in the sub-G1 phase (Fig. 2a and b), increased the levels of the cleaved forms of caspases 3, 7, and PARP, and decreased the levels of pro-caspases 3, 8, and 9 (Fig. 5a and b). Further, 21-900 induced G2/M arrest and tubulin depolymerisation that is consistent with the effect of vincristine. These results suggest that 21-900 treatment prevented the formation of normal spindle microtubules, leading to G2/M arrest. Further, the expression of proteins that mediate M phase was evaluated to identify the phase of cell-cycle arrest. Among them, Cdc25c is a tyrosine phosphatase that removes the inhibitory phosphorylation of Cdc2 at Tyr15, which has already been phosphorylated on Thr161 and contributes to Cdc2 activation, leading to the formation of the Cdc2/cyclin B complex and the transition of cells to M phase^21^. We show here that 21-900 decreased the levels of p-Tyr15 Cdc2, and increased the levels of cyclin B, aurora A, PLK1, p-Thr210 PLK1, and mitosis-specific phosphorylated MPM2 (Fig. 4a and b). The pan-HDAC inhibitor SAHA does not increase an MPM2 level^16^, which indicates that 21-900 induced G2/M phase arrest through the induction of microtubule dynamics instability but did not inhibit HDACs. A previous study demonstrated that Cdc2/cyclin B activates BCL-2 phosphorylation in response to MTAs, causing G2/M arrest that is followed by BAX activation, which mediates the apoptotic permeabilisation of the mitochondrial outer membrane^22^. It was reported that microtubule targeting agents evoked cell undergoing apoptosis were differentially regulated by mitogen-activated protein kinase (MAPKs) dependent on cell types^23^. Among mammalian MAPKs, JNK have been considered as a stress inducible kinase on apoptosis. Furthermore, Yamamoto *et al*. have found that the apoptosis signal-regulating kinase 1 (ASK1)/JNK pathway can be activated at G2/M phase, leading to BCL-2 phosphorylation in both normally cycling cells and paclitaxel treated cells^24^. Sustained BCL-2 hyperphosphorylation was linked to apoptosis by preceding caspase-3 cleavage^25^. Therefore, to determine whether JNK activation was involved in 21-900-induced apoptosis, we used the JNK inhibitor SP600125. As a result of Fig. 6a and b, 21-900, SAHA and vincristine all can induce JNK phosphorylation. It has been reported that JNK activation plays a vital role in microtubule binding agents triggered apoptosis^23^. Nevertheless, SAHA induced apoptosis of HL-60 cells is mediated by inactivation of ERK signaling pathway^26^. In our study, we found that JNK inhibitor, SP600125 can dramatically rescue 0.3 μM 21-900 reduced cell viability; by contrast, only partial response on 1 μM (Supplemental Fig. 3a), at which concentration inducing H3 acetylation (Fig. 1e). These results indicated that at lower concentration (0.3 μM), 21-900 can induce cell apoptosis though JNK activation, which was contributed by microtubule dynamic changes. However, the apoptotic effect of 21-900 at higher concentration (1 μM) was mediated by both microtubule deploymerization and HDAC inhibition pathways. SP600125, in the presence of 21-900, prevented BCL-2 phosphorylation and the cleavage of pro-caspase-3 (Fig. 6e and f). These findings demonstrate that in cells treated with 21-900, activated JNK phosphorylated BCL-2, leading to the induction of arrest of the cell cycle in M phase, which led to apoptosis.

These findings support the conclusion that the new synthetic agent 21-900 inhibited HDAC activity and caused microtubule depolymerisation, leading to cell-cycle arrest in M phase, JNK activation, and BCL-2 phosphorylation, which contributed to apoptosis. Moreover, treatment with 21-900 significantly inhibited the growth of engrafted HL-60 and MOLT-4 cells in nude mice without causing the mice to lose body weight. We conclude, therefore, that 21-900 has potential for use as a new drug candidate for cancer therapy.

## Methods

### Cell lines

Human acute myeloid leukemia cell line HL-60, human T cell acute lymphoblastic leukemia cell line MOLT-4, and human chronic myeloid leukemia cell line K562 were purchased from American Type Culture Collection (ATCC, Manassas, VA, USA). HL-60 and K-562 were maintained in Iscove’s modified Dulbecco’s medium (IMDM) with 10% or 20% fetal bovine serum, penicillin (100 units/ml) and streptomycin (100 μg/ml); MOTL-4 cells were cultured in RPMI-1640 with 10% fetal bovine serum, penicillin and streptomycin. All cells were incubated in the presence of 5% CO_2_ at 37 °C.

### Chemicals and antibodies

Vincristine, propidium iodide, anti-β-tubulin, FITC-conjugated anti-mouse IgG, poly-L-lysine hydrobromide, and SP600125 were purchased from Sigma Chemical Co. (St. Louis, MO, USA). Suberoylanilide hydroxamic acid (SAHA) and 21-900 (HDAC/tubulin inhibitor) were synthesized by Dr. Jing-Ping Liou (School of Pharmacy, College of Pharmacy, Taipei Medical University, Taiwan). The above drugs were dissolved in DMSO (dimethylsulfoxide) and then stored at −20 °C. The acetyl-Histone H3, cdk1, caspase 3, PLK1 (p-Thr210), cyclin B1, cdk1/cdc2 (p-Tyr15), and caspase 7 antibodies were all purchased from BD Biosciences (San Jose, CA, USA). Caspase 8, PARP, MPM2 (pSer/pThr), PLK1, and α-actin were purchased from Millipore (Bedford, MA, USA). Aurora A, p-JNK, BID, BCL-2, p-BCL-2, MCL-1, BAX, BAK, Ac-α-tubulin, caspase 9, and cleaved caspase 3 were purchased from Cell Signaling Technologies (Beverly, MA, USA). The labeled secondary antibodies goat anti-mouse IgG-HRP and goat anti-rabbit IgG-HRP were purchased from Santa Cruz (Santa Cruz, CA, USA).

### MTT assay

Cell viability was measured by MTT assay. Cells (3 × 10^5^ cells/well, 1 ml culture medium) were seeded in a 24-well plate and treated with different doses of 21-900 for 48 h. Cell proliferation was determined by incubating the cells with 100 μl of 3-(4,5–dimethylthiazol–2–yl)-2,5-diphenyltetrazolium bromide (MTT) (Sigma-Aldrich) solution for 1 h at 37 °C. After removal of the MTT solution, the resulting formazan crystals were dissolved in dimethyl sulfoxide and the plates were read by measuring the absorbance at 550 nm using a microplate reader (Molecular Devices, Sunnyvale, CA, USA).

### Flow cytometry

After drug treatment, cells were collected and washed with cold PBS, and then fixed with 75% alcohol overnight at −20 °C. After centrifugation, the fixed cells were washed with cold PBS, resuspended in a DNA extraction buffer (0.2 M NaPO_4_, 0.1 M citric acid, pH 7.8) for 30 min. The cells were centrifuged and stained with propidium iodide staining buffer (0.1% Triton X-100, RNase A and propidium iodide) for 30 min. Cell-cycle distribution was analyzed by FACScan Flow cytometer and Cell Quest software (Becton Dickinson, Mountain View, CA, USA).

### Immunofluorescence staining

Microtubule distribution and morphology were detected by immunofluorescence. 12 mm cover slides were placed in the 24-well plate and coated with poly-L-lysine hydrobromide for 1 h to enhance the suspension cells attached to the cover slides. Cells were seeded into the 24-well (8 × 10^5^ cells/well) and treated with 21-900, vincristine, and Taxol for 12 h. The following experiments were performed at room temperature. The cells were fixed with 8% paraformaldehyde in PBS for 15 min. After being washed twice (10 min/time) with PBS, the cells were permeabilized with 0.1% Triton X-100 in PBS for 10 min. Then, the cells were rinsed two times with PBS for 10 min. For blocking, 3% BSA in PBS was used. After 1 h, the cells were washed with PBS and incubated with a primary β-tubulin antibody (1:200) overnight, and then incubated with FITC-conjugated anti-mouse IgG antibody (1:200) for 2 h. The mounting medium, which contains DAPI stain, was dropped onto the slides, and cover slides were recovered to the slides. Images were detected and captured with the ZEISS LSM 510 META confocal microscope.

### Western blotting

Cells were incubated for 10 min at 4 °C in lysis buffer (50 mM Tris, pH 7.4; 150 mM NaCl; 1% Triton X-100; 1 mM EDTA; 1 mM EGTA, 1 mM PMSF, 10 μg/ml aprotinin, 10 μg/ml leupeptin, 1 mM sodium orthovanadate, and 1 mM NaF) for 30 min, and then centrifuged at 14,000 rpm at 4 °C for 30 min. Total protein content was quantified by BCA Protein Assay Kit (Thermo Fisher Scientific, Rockford, IL, USA). The whole-cell extract was mixed with 5 × sample buffer (312.5 mM Tris pH 6.8, 10% SDS, 50% glycerol, 0.05% bromophenol blue, 10% 2-mercaptoethanol) under heat at 95 °C for 10 min. Equivalent aliquots of proteins were electrophoresized on SDS-PAGE and subsequently transferred to PVDF membrane. The membranes were then blocked with 5% non-fat milk for 1 h and incubated with specific antibodies in PBST buffer overnight at 4 °C, followed by incubation with the appropriate HRP-conjugated secondary antibody. The membrane was washed frequently with PBST. Images of the Western blots were visualized and recorded by the chemiluminescence detection system (Amersham, Buckinghamshire, UK).

### Tubulin polymerization assay

Assays were performed according to the manufacturer’s instructions of cyto DYNAMIX Screen kit (Cytoskeleton Inc., Denver, CO, USA). Tubulin protein (10 mg/ml), GTP stock (100 mM), and general tubulin buffer were prepared following the protocol. Before detection, a 96-well plate was put on the spectrophotometer to pre-warm at 37 °C. Then the tubulin polymerization buffer, tubulin stock, and indicated drug were gently mixed. Finally, the mixture was transferred to pre-warmed plates. The absorbance was measured by a Spectra Maxplus ELISA reader (Molecular Devices, Sunnyvale, CA, USA) and recorded every 1 min for 30 min at 340 nm and 37 °C.

### Immunohistochemistry analysis

The tumor tissues from mice were washed with 1 × PBS. then fixed with 10% neutral buffered formalin, and embedded in paraffin using standard protocols. Then, tissue slices were deparaffinized in xylene and rehydrated in a graded series of ethanol. Slides were soaked to block endogenous peroxidase with 3% H_2_O_2_. For antigen retrieval, tissue slices were heated in 95–100 °C Tris-EDTA Buffer (101 mmol/L Tris Base, 1 mM EDTA solution, 0.05% Tween 20, pH9.0) for 30 min. The sectioned slides were incubated with a diluted primary antibody, Ac-Histone H3 (BD Bioscience San Jose, CA) or cleavage caspase 3 (Cell Signaling, Beverly, MA), or stained with hematoxylin and eosin. After rinsing with PBS several times, the secondary antibody, HRP polymer conjugate reagent A was applied to the slices, and subsequently reagent B for peroxidase catalyzation, which distinguishes the site of antigen. In each incubation step, the slides were followed by washing with PBS for 5 min. Then, Mayer’s Hematoxylin solution was used for counterstaining. The color of nuclei of cells was blue, and cytoplasm was pink. It could help to distinguish the nuclei and cytoplasm of tumor cells. The quantifications of Ac-Histone H3 and cleavage caspase 3 expression were analyzed by HistoQuest software (TissueGnotics GmbH, Vienna, Austria).

### *In vivo* xenograft model

*In vivo* tumor xenograft model was usually used to estimate the antitumor activity of drugs. Thirty female SCID mice were 4 weeks old, and were established by a subcutaneous injection of 1 × 10^7^ cells/ml with leukemic cells (HL-60 and MOLT-4). When the average tumor size reached 500 mm^3^, mice were separated to three groups (one group for 5 mice) and then treated with an indicated dosage of 21-900 by oral administration, one a day (qd). Tumor size and body weight were measured twice a week for the duration of the experiment. Tumor dimensions were measured using a caliper, and the volume was calculated using the following formula: volume (mm^3^) = width^2^ × length × 0.5. At the end of the study (average size of the tumor was greater than 2,500 mm^3^), mice were sacrificed and tumors were removed, weighed, and frozen in formalin for immunohistochemical analysis and the western blot experiments to evaluate the action of drugs *in vivo*. Animal experiments were performed in accordance with relevant guidelines and regulations followed ethical standards, and protocols has been reviewed and approved by Animal Use and Management Committee of Taipei Medical University (IACUC number: LAC-2013-0139).

### Data Analysis and Statistics

Each result represents a Mean ± SEM of at least three independent experiments that were analyzed using Student’s *t-test*. One-way ANOVA was conducted in animal study. Parameters with *p*-value < 0.05 are considered statistically significant.

## Additional Information

**How to cite this article:** HuangFu, W.-C. *et al*. Anti-leukemia effects of the novel synthetic 1-benzylindole derivative 21-900 *in vitro* and *in vivo. Sci. Rep.* **7**, 42291; doi: 10.1038/srep42291 (2017).

**Publisher's note:** Springer Nature remains neutral with regard to jurisdictional claims in published maps and institutional affiliations.

## Supplementary Material

Supplemental Figures
