# Supplementary material for: Anti-leukemia effects of the novel synthetic 1-benzylindole derivative 21-900 in vitro and in vivo
Source: Sci Rep. 2017 Feb 9;7:42291. doi: 10.1038/srep42291 (PMC5299419; doi:10.1038/srep42291)
Supplement: Supplemental Figures [file srep42291-s1.pdf]

Anti-leukemia effects of the novel synthetic 1-benzylindole derivative 21-900 *in vitro* and *in vivo*

Wei-Chun HuangFu, Min-Wu Chao, Chun-Chun Cheng, Yu-Chieh Wei, Yi-Wen Wu,
Jing-Ping Liou, George Hsiao, Yu-Ching Lee and Chia-Ron Yang

Supplemental Figure 1

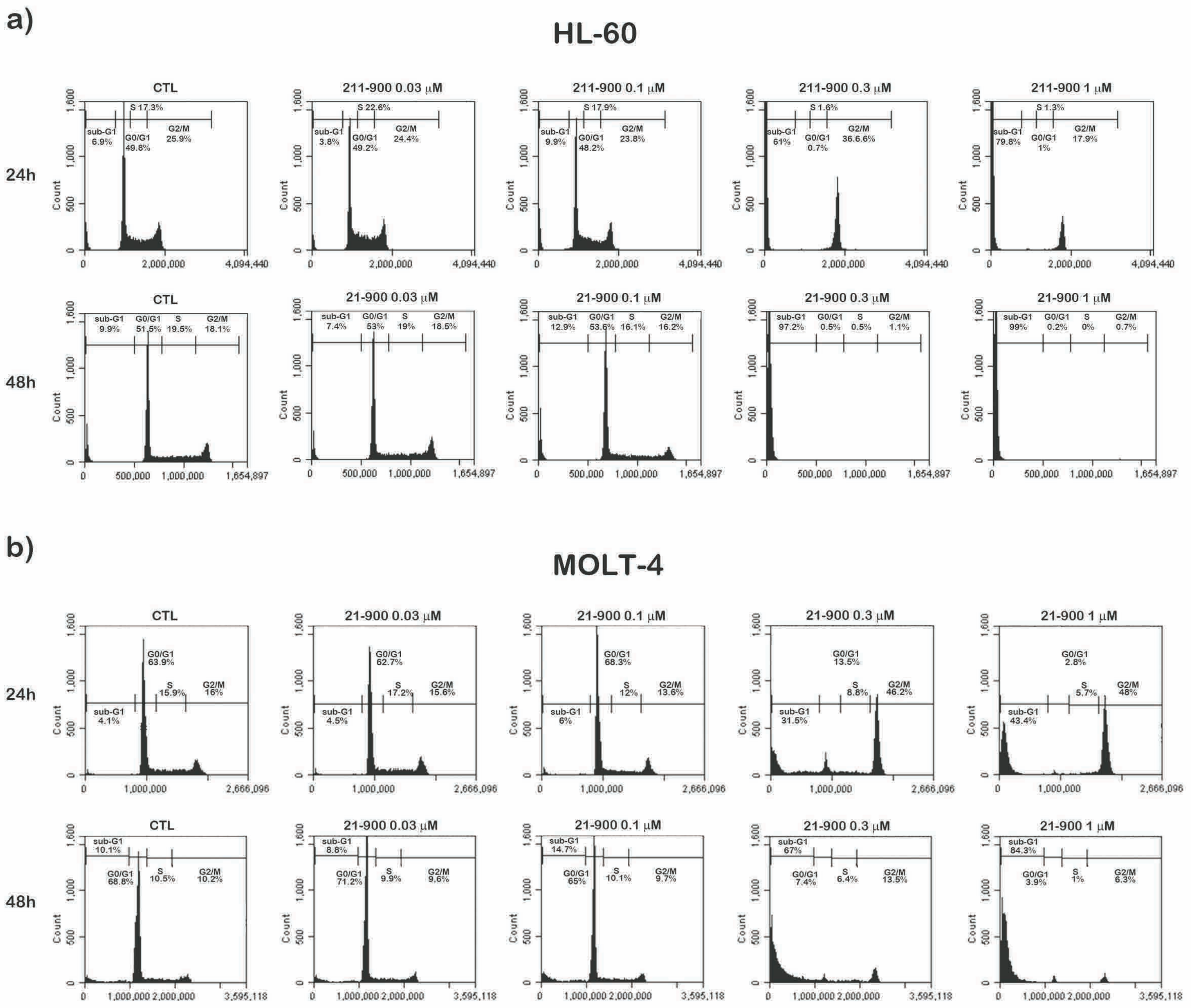

Supplemental fig. 1: The representative histograms of cell cycle distribution on HL60 and MOLT-4 cells in the presence of 21-900. (a) HL-60 or (b) MOLT-4 cells were treated with the indicated concentrations of 21-900 for 24 or 48 h, the cell cycle distribution was analysed using flow cytometry.

Supplemental Figure 2

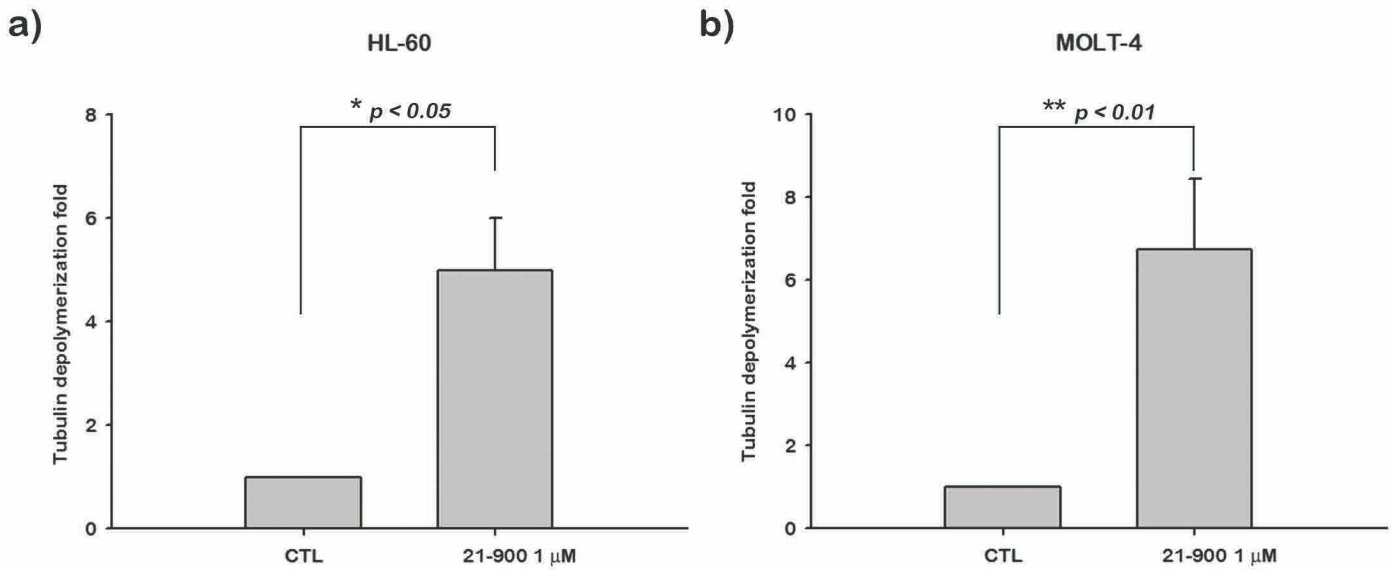

Supplemental fig. 2: 21-900 inhibits tubulin polymerization. The quantified results show the expression fold of tubulin depolymerisation in HL-60 (a) or MOLT-4 (b) cells after treatment of 24 h 12-900. The results represent the mean ± SEM at $^{*}p < 0.05$, $^{**}p < 0.01$ compared with control groups.

Supplemental Figure 3

a)

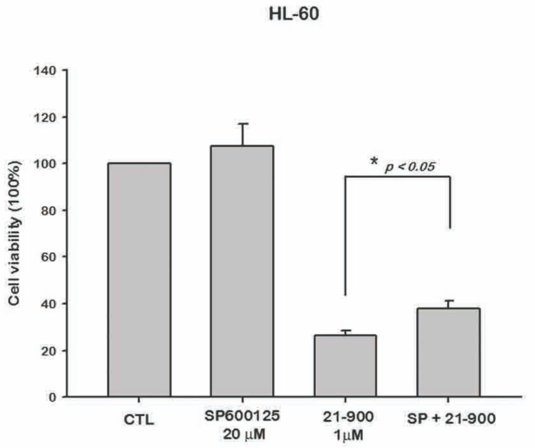

b)

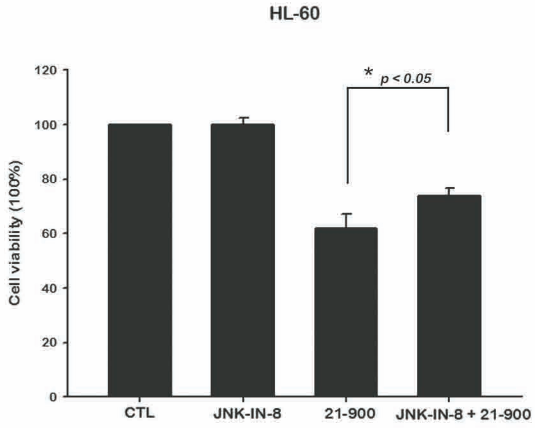

Supplemental fig. 3: 21-990 induced cell apoptosis can rescue in the presence of JNK inhibitor. (a) HL-60 cells were incubated with 21-900 (1 μM) with or without SP600125 (20 μM) for 48 h. (b) HL-60 cells were incubated with 21-900 (0.1 μM) with or without JNK-IN-8 (20 nM) for 36 h. Cell viability was measured by using the MTT assay. The results represent the mean ± SEM at $*p < 0.05$ compared with indicated groups.

Supplemental Figure 4

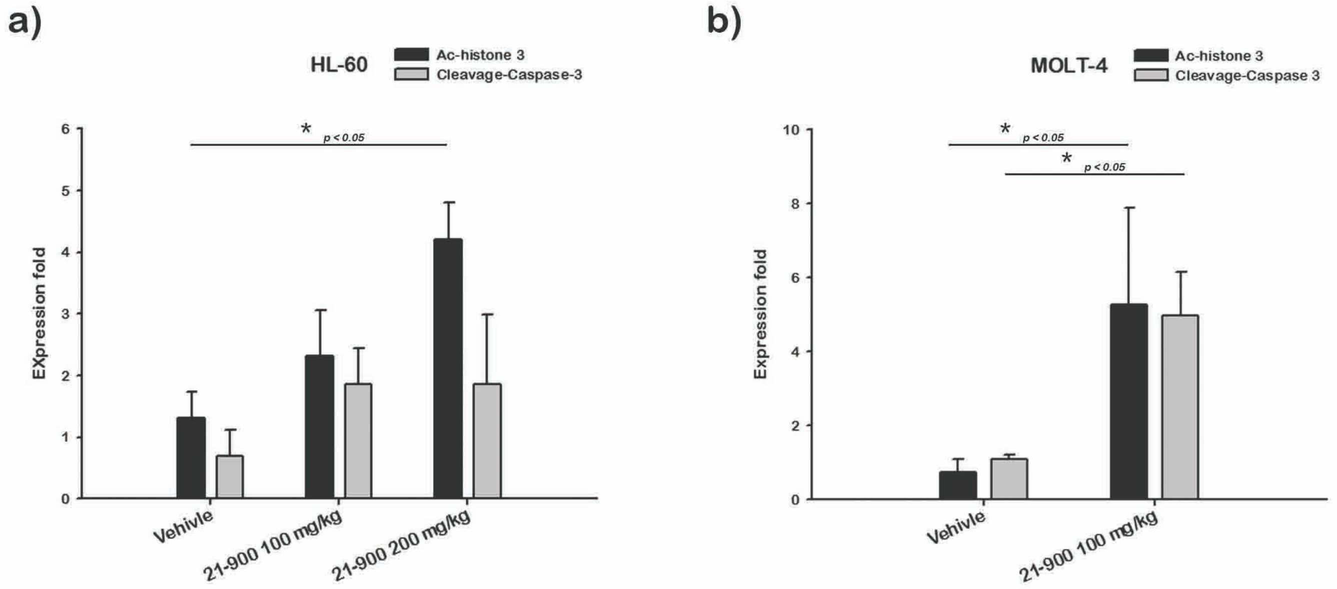

Supplemental fig. 4: 21-900 induces acetyl-histone H3 and cleavage caspase 3 expression in human leukaemia xenograft model. Quantification of immunohistochemistry stained in acetyl-histone H3 and cleavage caspase 3 expression. Results represent the mean ± SEM at *$p < 0.05$ compared with vehicle groups.
